# The Vega advanced third generation posterior stabilized total knee arthroplasty system enables the restoration of range of motion for high demanding daily activities – A 5-years follow-up study

**DOI:** 10.1371/journal.pone.0302885

**Published:** 2024-05-13

**Authors:** Ludger Gerdesmeyer, Claudio Glowalla, Igor Lasic, Munjed Al Muderis, Matthias Weuster, Tim Klueter

**Affiliations:** 1 Department of Orthopaedic Surgery, Mare Klinik, Kiel-Kronshagen, Germany; 2 Department of Orthopaedics and Sportorthopaedics, Klinikum Rechts der Isar, München, Germany; 3 Macquarie University Hospital, Macquarie University, Sydney, Australia; 4 Department of Orthopaedics and Traumatology, DIAKO Hospital, Flensburg, Germany; 5 Department of Orthopaedic Surgery and Traumatology, University of Kiel, Kiel, Germany; IRCCS Istituto Ortopedico Rizzoli, ITALY

## Abstract

**Background:**

The Vega System® PS (Aesculap AG, Tuttlingen, Germany) is an advanced, third generation fixed implant that aims to mimic natural knee kinematics by optimizing pivotal motion while reducing surface stress. This study evaluated mid-term survival and clinical outcomes, including range of motion (ROM) of the modern posterior stabilized implant in order to analyse whether this biomechanically successful implant reaches good results in situ.

**Methods:**

The first 100 patients to receive the Vega PS System for total knee arthroplasty were invited to take part in this single centre, single surgeon study. Of these, 84 patients were clinically assessed 5–6 years postoperatively. Data which was obtained during this follow-up examination included revision data, range of motion and clinical scores.

**Results:**

The 5-year survival rate for exchange of any component was 97.6%, whereby two patients required replacement of the polyethylene gliding surface. Secondary patella resurfacing was performed in 7 patients. Significantly improved results in comparison to the preoperative state could be obtained at the follow-up: KOOS improved from 39.4 to 78.8, SF-12 PCS improved from 32.1 to 42 SF-12 MCS improved from 46 to 53.8 and patella pain improved from 2.7 to 0.3. The mean ROM of the 84 patients after 5 years was 133.1° and mean total KSS was 189.9.

**Discussion & conclusions:**

This study demonstrates a high survival rate of the Vega PS System® and significant improvements in clinical outcomes 5 years after implantation. The obtained mean ROM indicates that this implant provides good flexibility of the knee joint, allowing a high number of activities. However, due to the rate of secondary patella implantation, routine resurfacing of the patella for all PS TKA cases is highly recommended.

**Clinical trials registration:**

The study was registered at clinicaltrials.gov (NCT02802085).

## Introduction

Total knee arthroplasty (TKA) is a common and highly effective intervention for the treatment of severe knee joint conditions where conservative therapies have not shown improvements in patient outcomes. The principal indications are mainly degenerative osteoarthritis (>95%), but also rheumatoid or posttraumatic arthritis, osteonecrosis, symptomatic knee instability, stiffness or deformation of the knee joint [1]. Rapidly growing implantation rates are mainly due to an aging population [2] and growing prevalence of obesity [3].

Several modern TKA implant designs are available, including cruciate-retaining and posterior-stabilized (PS) designs. The latter requires resection of the posterior cruciate ligament. The use of PS TKA implants is rising in the German Arthroplasty Registry (EPRD) from 16.6% in 2015 to 25.6% in 2022 [3], whereas it is decreasing in the National Joint Registry of England and Wales from 21.2% in 2010 to 15.3% in 2022 [4] and in the Australian Orthopaedic Association National Joint Replacement Registry (AOANJRR), where PS TKA accounted for only 13.3% of all primary TKA [5].

PS TKA implants showed better kinematics and postoperative flexion ability compared to CR TKA implants [6–8], although the impact on the daily life of patients is still a matter of debate. The Vega System® PS is an advanced, third generation fixed implant that aims to mimic natural knee kinematics by optimizing pivotal motion while reducing surface stress. So far, 3-dimensional and biomechanical data [9–11], intraoperative analyses [12, 13] and short-term clinical results [14–20] up to 3 years of this implant were published. The aim of this study was to fill the gap and collect mid-term functional and clinical data of the Vega System® PS TKA implant.

## Materials and methods

### Study design

**Fig 1:**
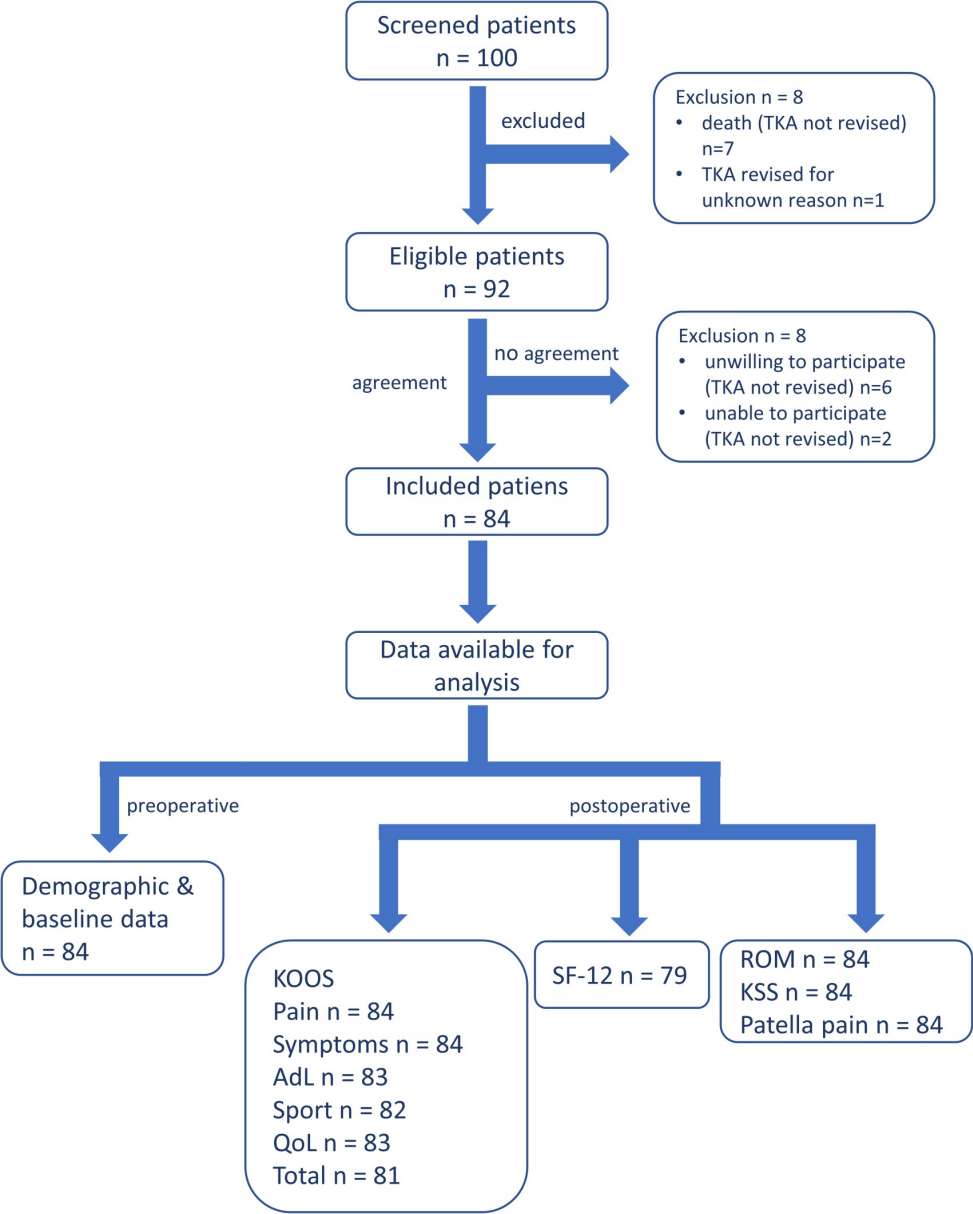
Patient inclusion flow diagram.

The first one-hundred patients (n = 100) who had a TKA at the study site (MEDBALTIC GmbH, MARE Klinikum, Kiel, Germany) with the use of the Vega System® PS (Vega System® PS, Aesculap AG, Tuttlingen, Germany) were invited to participate in this prospective single arm, single center, single surgeon cohort study, which evaluated clinical and functional outcomes 5 to 6 years after implantation. Eighty-four patients (n = 84) who were in compliance with the inclusion/exclusion criteria gave their informed consent. The remaining 16 patients did not participate for the following reasons: unwilling to return for the 5-year postoperative follow-up examination, TKA not revised (n = 6); death, TKA not revised (n = 7); unable to return for the 5-year postoperative follow-up examination, TKA not revised (n = 2); TKA revised for unknown reason (n = 1) (see Fig 1).

Study inclusion criteria were primary TKA at the study site 5 to 6 years prior to enrolment using the product under investigation and ≥ 18 years of age at the time of surgery, and written informed consent of the patient. Primary TKA was performed for severe knee joint conditions that could not be treated through other therapies, including degenerative osteoarthritis, rheumatoid arthritis, posttraumatic arthritis, symptomatic knee instability, knee stiffness or deformation of the knee joint. Study exclusion criteria were according to the instructions for use of the implant system: patients for whom reconstructive surgery to treat the joint disorder is an option, acute or chronic infections near the joint, secondary diseases influencing the function of the joint implant, bone tumours in the region of implant fixation, poor bone quality and osseous malformations, diseases in the area of the implant fixation, which may primarily or subsequently affect the stability of the joint replacement anchorage, known hypersensitivity to the implant materials.

The study was registered at clinicaltrials.gov (NCT02802085). The study received ethical approval on 2016-04-28 (internal number: 048/16 II;) from the responsible ethical review board (Ethikkommission bei der Landesärztekammer Schleswig-Hostein (Bad Segeberg, Germany)). All patients gave written informed consent prior to study enrolment. The recruitment period for this study started on 2017-01-11 and ended on 2017-11-27. A filled STROBE statement checklist is provided as S1 Checklist.

### Device description

**Fig 2:**
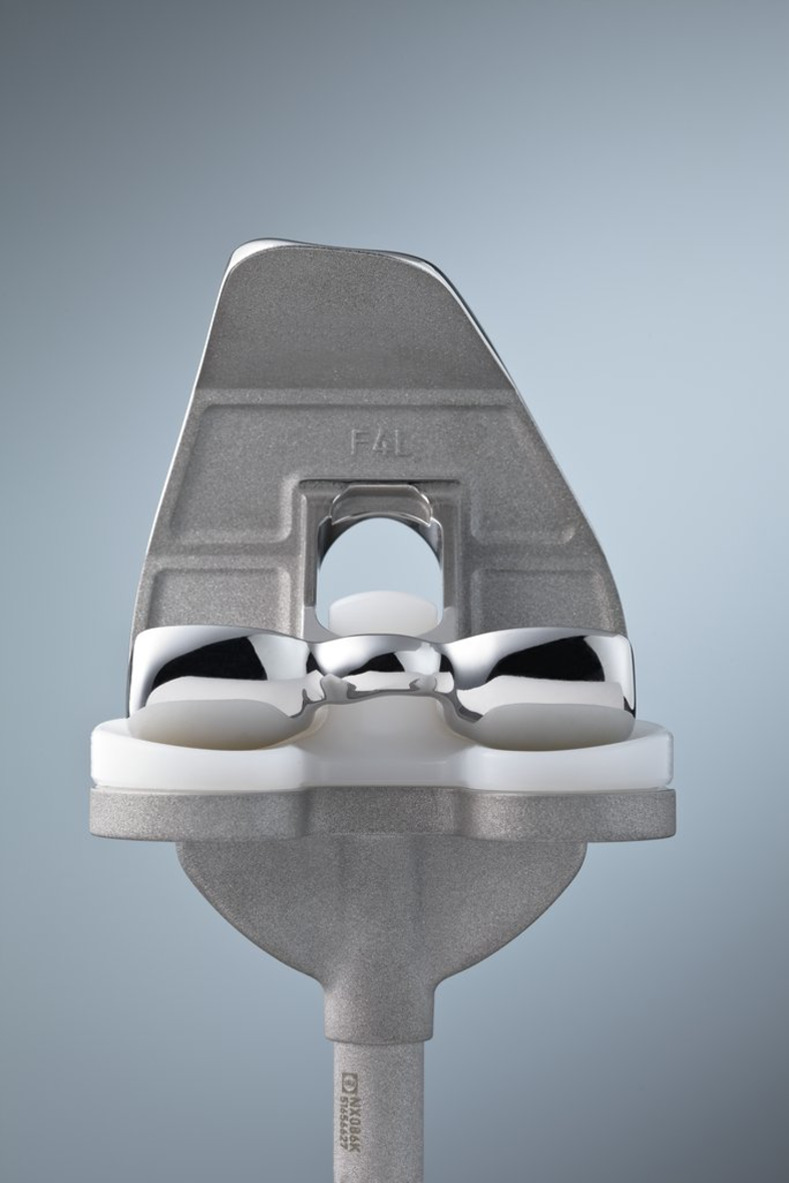
Vega System® in cobalt-chromium.

The Vega System® PS is an advanced, third generation fixed implant that aims to mimic natural knee kinematics by optimizing pivotal motion while reducing surface stress. Its asymmetric cam design facilitates the physiological rollback of the lateral femoral condyle with simultaneous rotation of the medial condyle (see Fig 2). The high congruency between femur and tibia in extension as well as the line contact up to 160° of flexion [21], aim to further stabilize the patient’s movement and to reduce wear. The Vega System® PS is available in cobalt-chromium and in a multilayer ceramic surface version (Advanced Surface).

### Surgical procedure and postoperative management

Index surgeries were performed between November 30, 2010 and August 29, 2012. All surgeries were carried out with the standard cobalt-chromium implants by a single surgeon under general or spinal anesthesia using a tourniquet. Each patient received a single-shot of antibiosis and Tranexamic acid for antifibrinolysis. Medial parapatellar approach was performed in all cases. Patella was everted laterally, and the Hoffa fat pad was partially removed. Instrumentation of the Vega System® PS was used in the femur first technique, balancing adopted measured resection technique. The tibial cut was done including individual slope. Finally, patella plasty was done, osteophytes were removed as required followed by circumferential and retro-patellar electrocautery in most cases. Alternatively, patella resurfacing was performed on an individual basis. Cementing was performed in a single step with Gentamicin Palacos (Heraeus medical, Wehrheim, Germany) for all components. Wound closure was performed in layers with a low-vacuum drain left in place.

All patients were advised to undergo the same postoperative management including continuous passive motion as tolerated from the first postoperative day until discharge and immediate postoperative weight-bearing as tolerated. Full weight bearing and at least 90° of active flexion were a precondition for discharge. Antithrombotic prophylaxis was maintained for 6 weeks and patients were advised to undergo physiotherapy from the first postoperative day until 6 months.

### Outcome measures

Clinical outcomes obtained at the 5 years follow-up were:

- incidence of revision
- the total Knee injury and Osteoarthritis Outcome Score (KOOS) [22]
- the SF-12® Health Survey (SF-12) Physical Component Score (PCS) and Mental Component Score (MCS) [23]
- the Knee Society Score (KSS) with its Knee and Functional Score (KS and FS) [24]
- the anterior knee pain according to the Waters and Bentley rating system [25], consisting of four response categories ranging from grade 0 (no pain) to grade III (severe pain; patient considering further surgery) and is evaluated by the examiner asking the patient "Do you experience pain at the anterior aspect of the knee?".

ROM was assessed using a standard handheld goniometer to determine the maximum passive flexion under non-weight-bearing conditions and was calculated in a standardized manner within the KSS measurement. Furthermore, complications were recorded. Standard radiographs were taken at the 5-years examination including a/p long leg, lateral and 45° patella x-rays.

### Statistical analysis

Statistical analysis was performed using SAS software version 9.4 (SAS Institute Inc., Cary, NC, USA).

Although the objective of the study was to analyse the first 100 patients having received the implant, a power analysis was performed prospectively to figure out, whether clinically relevant effect can be proven with this sample size. In accordance with results of PS TKA implants in the Australian Orthopaedic Association National Joint Replacement Registry [4] at the time of calculation, a revision rate of 3.7% after 5 years was expected for the Vega System® PS. A revision rate of 15% after 5 years was set as non-acceptable limit, which is still below the rates of three failed PS TKA implants (revision rates ranging from 18.7% - 30.5%) in the registry report [4]. The study result has to be significantly below this 15% limit. A drop-out rate of 20% was expected, leaving 80 patients. An exact binominal test with a nominal 0.025 one-sided significance level will have 92% power to detect the difference between the null hypothesis proportion (p0 = 0.15) and the expected proportion (pa = 0.037) when the sample size is 80. This means that under these estimates, the power is more than sufficient for a standard study setting where power is typically set to 80%.

Survival was estimated using the Kaplan-Meier estimator. Cox proportional hazards model adjusted for patient age, sex and BMI was applied to analyse correlation between survival and these factors.

Multivariate linear regression models were applied to continuous outcome variables (KOOS, ROM, KSS) to analyse whether patient age, BMI or gender are influencing factors regarding these outcomes.

For comparisons between baseline and follow-up values of KOOS, SF-12 and patella pain, a paired t-test was used. Statistical significance was assumed at p<0.05.

## Results

### Patient demographics

Enrolled patients underwent surgery between November 30, 2010 and August 29, 2012. The primary diagnosis of all 84 patients was osteoarthritis of the knee. One patient presented with a condition after an anterior cruciate ligament repair. Table 1 summarizes patient demographics and baseline characteristics. In 3 of 84 patients (3.6%) the patella was resurfaced as those patients already had TKA of the contralateral side with patellar resurfacing and were satisfied with the result. No early postoperative complications were reported in any of the enrolled patients.

**Table 1:** Patient demographics and baseline characteristics.

	N (%)	Mean ± SD (Range)
**Index knee treated**	84 (100)	
**Left**	36 (42.9)	
**Right**	48 (57.1)	
**Sex**		
**Male**	35 (41.7)	
**Female**	49 (58.3)	
**Age, [years]**		65.1 ± 9.3 (26–81)
**Height**, **[cm]**		170.9 ± 9.7 (153–195)
**Weight, [kg]**		86.4 ± 17.17 (51–120)
**BMI**		
**normal (< 25)**	14 (16.7)	
**overweight (25–30)**	38 (45.2)	
**severe overweight (> 30)**	32 (38.1)	

### Survival

The mean postoperative follow-up time was 5.7 years (median 67.8 months; range 59.3 to 80.6 months; standard deviation (SD) 5.5 months). In two patients, the polyethylene gliding surface was replaced (one for instability and one in conjunction with a secondary patella resurfacing for anterior knee pain). Kaplan-Meier survival rate based on removal of any implant component (femoral, tibial or polyethylene replacement or removal) was 97.6% (95% confidence interval (CI) 90.8–99.4; last event month 55.2) at five years. No femoral or tibial revisions were performed.

Cox proportional hazards model adjusted for patient age, sex and BMI did not reveal any influences of these factors on survival of the implant.

### Clinical and functional outcomes

The total KOOS score, SF-12 PCS and MCS as well as the anterior patella pain rating according to Waters and Bentley improved significantly from preoperative baseline to 5-year follow-up (p<0.0001) (Table 2). KOOS sub-scale scores at 5 years postoperative are provided in S1 Table.

**Table 2:** Clinical outcome scores by visit.

		N	Mean ± SD (Range)	Score improvement (5-year FU–Baseline) [95% confidence limits]
**KOOS**	**Baseline**	84	39.4 ± 8.8 (19.0–55.0)	39.4 [34.9, 43.9]
	**5-year FU**	81	78.8 ± 18.6 (28.5–100.0)	
**SF-12 PCS**	**Baseline**	84	32.1 ± 3.1 (25.0–40.0)	9.8 [7.5, 12.1]
	**5-year FU**	79	42.0 ± 10.0 (17.5–57.0)	
**SF-12 MCS**	**Baseline**	84	46.0 ± 5.8 (31.0–56.0)	7.75 [5.6, 9.9]
	**5-year FU**	79	53.8 ± 8.1 (31.0–67.4)	
**Patella Pain Rating**	**Baseline**	84	2.7 ± 0.5 (1.0–3.0)	-2.4 [-2.6, -2.3]
	**5-year FU**	84	0.3 ± 0.5 (0.0–2.0)	

The ROM at the index knee, knee score (KS), functional score (FS) and total score at 5 years postoperatively are presented in Table 3.

**Table 3:** Five-year ROM and KSS sub-scale scores.

	N	Mean ± SD (Range)
**ROM**	84	133.1 ± 12.6 (95–150)
**KSS knee (KS)**	84	95.9 ± 5.9 (71–100)
**KSS functional (FS)**	84	94.0 ± 8.6 (55–100)
**KSS total**	84	189.9 ± 13.4 (126–200)

Multivariate regression modelling did not reveal any influences of patient age, gender or BMI on KOOS, ROM and KSS.

### Complications

At the 5-year follow-up examination, 8 patients (9.5%) reported complications related to the index knee (see Table 4). One patient presented with patellar arthrosis and pain which was not treated surgically and is currently ongoing. Re-operations were carried out in the other 7 patients (8.3%) who received a patella implant, due to painful retro-patellar arthritis. In one of those patients also synovectomy and arthrolysis was performed due to fibrosis. In further 2 of those 7 patients also the polyethylene gliding surface was replaced. One of those two cases presented with an infection and visible wear of the inlay.

**Table 4:** Complications at five years postoperatively.

Patient	Complication	Treatment (surgical)	Outcome
**1**	patellar arthrosis	patella replacement, exchange of inlay	resolved without sequelae
**2**	patellar arthrosis	patella replacement	resolved without sequelae
**3**	patellar arthrosis, postoperative infection with wear of the inlay	patella replacement, exchange of inlay	resolved without sequelae
**4**	patellar arthrosis, fibrosis	patella replacement, synovectomy, arthrolysis	resolved without sequelae
**5**	patellar arthrosis	patella replacement	resolved without sequelae
**6**	patellar arthrosis	patella replacement	resolved without sequelae
**7**	patellar arthrosis	patella replacement	resolved without sequelae
**8**	patellar arthrosis	n/a	ongoing

All patients completely recovered after surgery without sequela. Five years after index surgery, 5 of 7 patients with secondary patella replacement were without pain and the remaining 2 patients experienced mild pain which did not interfere with activities of daily living.

## Discussion

This study is the first to report mid-term survivorship and clinical outcome results for the advanced third generation PS TKA system. This 5-year follow-up study found good mid-term clinical and functional outcomes for the Vega System® PS TKA implant with a low rate of intra- and postoperative complications. The 5-year implant survival rate in this study was 97.6%. Clinical outcomes measured by KOOS, SF-12 and pain according to Waters and Bentley improved significantly compared to baseline values. More important, these improvements reach a clinically relevant effect for the patients according to a recent systematic review [26].

The mean ROM of the 84 patients after 5 years was 133.1 ± 12.5°. Previous reports of postoperative ROM for various PS TKA designs range from 94.3° to 133.6° [27–30]. The results from the current study show that a clear majority of patients (78.6%) reached at least 130° of flexion, and approximately half of the patients (52.4%) reached 140° or more, which allows for a wide range of demanding activities of daily living requiring deep knee flexion [31, 32]. Jain et al. [15] have reported similar results on mean maximum flexion measurements of 132.4° ± 10.4° for the Vega System® PS prosthesis as compared to 130.3° ± 11.1° for e.motion PS and 130° ± 13.8° for Genesis II at 2-year follow-up. In order to achieve the greatest functional results after TKA, a range of motion (ROM) of 128° to 132° is necessary, whereas ranges of less than 118° significantly diminish positive outcomes [33]. Squatting, kneeling or sitting cross-legged are activities which are more frequently performed in Asia and the Middle East. However, they are becoming more frequent in the western world, whether for religious reasons or in the context of leisure activities (yoga, meditation). Performing these activities requires 111° to 165° (or full) knee flexion [31, 32]. It has been shown that a minimum of 110° of flexion is a suitable goal to restore motion in the knee [34], although other reports indicate that on average knee patients rarely flex beyond 120° after TKA [35].

Two patients required replacement of the polyethylene gliding surface resulting in 97.6% implant survival rate for exchange of any components. Secondary patella resurfacing was performed in both revision surgeries. Five additional patients required secondary surgery due to progressing painful retro-patellar arthritis with subsequent patella implantation resulting in a total 5-year reoperation rate of 8.3% (95% CI 4.1–16.7) due to any reason. For PS TKA without patellar resurfacing, revision rates of 5.4% (95% CI 5.2–5.7) were reported in the AOANJRR [5], whereas revision rates of PS TKA implants with patellar resurfacing at the same time were 3.1% (95% CI 3.0–3.2). Nizard et al reported in their meta-analysis pooled patella reoperation rates of 6.5% (range 0%-15%) and anterior knee pain rates ranging from 5.8% up to 30.7% for the non-resurfaced group (based on 12 Randomized Controlled Trials (RCT)); and 7.9% (range 4.8%-11.8%) based on the three-best quality RCTs in the non-surfaced patella group [36]. Although the rate of secondary patella surgery in this study is within the range reported by other authors, particular attention should be paid to this occurrence of clinical relevance. In the present study, for all patients who required subsequent patella implantation due to severe pain, the issue was remedied after secondary surgical treatment. Five years after initial surgery, 5 of 7 affected patients were pain free and two patients experienced mild pain which did not impact daily activities.

In clinical practice, PS implant designs have been associated with a higher likelihood of secondary patellar resurfacing. The more restricted movement of PS systems may be related these observations, but biomechanical explanations in the literature are rare [37]. It has been shown that a PS design, compared to a medially stabilized design, results in higher retropatellar facet pressure [38], explaining the increased likelihood of secondary patellar resurfacing also found in this study. On the other hand, biomechanical testing showed no increased retropatellar pressure of the Vega System® PS TKA in comparison to a CR TKA implant [39]. In that study the authors hypothesized that the external tilt of the PS implant creates an altered pressure point with more strain on the patella facets, causing increased pain in patients and resulting in increased rates of patellar resurfacing probability in the EPRD [39].

In general, third generation PS TKAs have been designed to provide patients with deep knee flexion in combination with knee stability, even for unstable knees, during high demanding activities while maintaining survivorship rates to those of standard TKA designs.

For patients with a weak posterior cruciate ligament, the posterior-stabilized Vega System® PS can support the medial pivoting and rollback of the native knee without enforcing it [40]. With the TKA system, individual rotational patterns were found to be preserved. The native femoral rollback is maintained or even increased and on average, the Vega PS design follows the native kinematics more closely, compared to other designs [41].

The fact that PS knees show more rollback compared to cruciate retaining TKAs and less than native knees has also been described by Dennis et al. [42]. However, the near-native rollback reported for the Vega PS design [41] could not be observed in any of the PS systems investigated by Dennis et al. This rollback should prevent any posterior impingement and thereby allow a high flexing and good kinematic function of the knee which could be an explanation for the good ROM results achieved in this study.

There are limitations to this study, which may be a source of bias, that should be considered. First, this consecutive, non-randomized cohort design lacks a control group. However, comparisons from reports in the literature are intended to alleviate this weakness. Second, not all of the 100 first consecutive implantations could be included into the study and therefore information on the implant status and functional outcome of these patients is not clear. This may cause attrition bias, as there is the possibility that the group of patients not participating in the study differs from the group of patients included in the study. Nevertheless, survivorship information was obtained for those 16 patients who belong to the cohort but did not give their consent to participate in the study. In one of these patients the prosthesis has been revised externally for reasons unknown to the author. Third, patients from a single centre were included in this study, introducing a possible source of selection bias. Still, baseline data of our patient group is comparable to patients in other TKA studies published in the literature and in implant registries [3, 5, 43]. Fourth, the sample size of this study is not based on a priori sample-size calculation considering the hypothesis of the study. Rather, the study sample included the first 100 patients treated by the corresponding author with this implant. Still, a prospective power analysis indicated that this sample size is sufficient to allow a statistically sound statement regarding the study hypothesis. Finally, the study does not address longer-term outcomes beyond 5 years postoperative. Although no long-term results are yet available for the TKA system under investigation, the mid-term results obtained herein can provide initial indications of clinical efficacy and safety under routine conditions. A strength of the study is that all surgeries were performed by a single surgeon according to a standard operative and postoperative routine, limiting a possible performance bias. Also, reporting bias is limited as all recorded data and scores are included in this manuscript and provided as raw data.

## Conclusions

The study results demonstrate that the advanced third generation Vega System® PS TKA significantly improves clinical outcomes 5 years after implantation and enables most patients to perform demanding activities of daily living that require 130° of flexion or even more. Due to the progressive rise of femoro-patellar joint forces in deep flexion, resurfacing the patella in all PS TKAs is recommended as a matter of routine to avoid subsequent patella implantations. The 5-year survival rate does not indicate increased implant related risks such as aseptic loosening or instability. Nevertheless, further comparative studies with a suitable number of cases are required to obtain evidence based longer-term results.

## Supporting information

S1 Checklist STROBE checklist. (DOCX)

S1 Table Five-year KOOS sub-scale scores. (DOCX)

S1 Raw data (XLSX)

## List of abbreviations

AOANJRR: Australian Orthopaedic Association National Joint Replacement Registry
BMI: Body-mass index
CR: Cruciate retaining
EPRD: Endoprothesenregister Deutschland (German Arthroplasty Registry)
FS: Functional score
KOOS: Knee Injury and Osteoarthritis Outcome Score
KS: Knee score
KSS: Knee society score
MCS: Mental component score
PCS: Physical component score
PS: Posterior stabilized
ROM: Range of motion
SD: Standard deviation
SF-12: Short Form Health Survey
TKA: Total knee arthroplasty
