# Supplementary material for: The Vega advanced third generation posterior stabilized total knee arthroplasty system enables the restoration of range of motion for high demanding daily activities – A 5-years follow-up study
Source: PLoS One. 2024 May 13;19(5):e0302885. doi: 10.1371/journal.pone.0302885 (PMC11090364; doi:10.1371/journal.pone.0302885)
Supplement: S1 Table — (DOCX) [file pone.0302885.s002.docx]

| \| Supplementary Table 1: Five-year KOOS sub-scale scores. \| \| --- \| |
| --- | --- |
|  |
| \| \|  \| **N** \| **Mean ± SD (Range)** \| \| --- \| --- \| --- \| \| **KOOS pain** \| 84 \| 85.6 ± 16.3 (36.1-100) \| \| **KOOS symptoms** \| 84 \| 85.0 ± 13.0 (50.0-100) \| \| **KOOS ADL** \| 83 \| 85.7 ± 16.7 (31.3-100) \| \| **KOOS sport** \| 82 \| 65.0 ± 30.4 (0-100) \| \| **KOOS QoL** \| 83 \| 71.8 ± 26.6 (0-100) \| \| **Total KOOS** \| 81 \| 78.8 ± 18.6 (28.5-100) \| \| \| --- \| --- \| --- \| --- \| --- \| --- \| --- \| --- \| --- \| --- \| --- \| --- \| --- \| --- \| --- \| --- \| --- \| --- \| --- \| --- \| --- \| --- \| |
